# Enter Preprint Servers: Is Peer Review Obsolete?

**DOI:** 10.1016/j.shj.2023.100207

**Published:** 2023-07-14

**Authors:** Anthony DeMaria

**Affiliations:** Judy and Jack White Chair in Cardiology, Sulpizio Cardiovascular Center, University of California, San Diego, La Jolla, California, USA

The delay in the publication of new information is the critique of medical journals that has received the most recent and robust attention. Fueled by the need to rapidly transmit new findings during the Covid pandemic, the time required to provide peer review and ultimately make new data available to readers has been the focus of intense scrutiny. The result has been the development of preprint servers, which provide the ability to post the results of completed research manuscripts online and available to the public. The most prominent of these servers for medicine is MedRxiv organized by Yale University in conjunction with Cold Spring Harbor and the British Medical Journal.

Space does not allow complete review of preprint servers as is available in other reviews.^2^ They clearly convey some significant benefits compared to current medical publications. The greatest benefit is the rapid dissemination of new research findings. Enthusiasts point out that the process of selecting reviewers and receiving critiques, the evaluation and priority assignment by the editors, the performance of any requested additional data or required revisions, the forwarding of accepted manuscripts to the publishers for copy editing, typesetting, and ultimate publication online or in press necessitate a substantial amount of time. The data are not available to the public during this process, and perhaps for a longer duration to many if restricted only to subscribers. Preprint servers eliminate this interval and rapidly and widely disseminate the new findings. In addition, the availability of preprints provide the possibility of receiving and incorporating constructive critiques prior to journal submission. Finally, the appearance in a preprint server enables the authors to stake credit for originality.

The most obvious limitation of preprint servers is the potential to circulate inaccurate information. The danger of disseminating erroneous information is amplified if propagated widely in the press, online, and in social media. The findings reported in the medical literature are susceptible to misinterpretation or exaggeration by the media, and this potential appears to be increased for preprint servers. The final manuscript submitted to and published in a journal may differ significantly from that posted on the server. While the most reputable preprint sites stress that the data have not been peer reviewed and is not actionable, this contrasts somewhat with the need for rapid dissemination.

As delineated in an earlier essay, the origin of peer review is usually credited to Henry Oldenburg who, as Editor of the Philosophical Transactions of the Royal Society, was the first to send out submissions for independent external evaluation.^1^ He thereby initiated the practice of peer review, which is the foundation for current medical journals. This practice has been periodically praised and criticized since that time. It appears we are now undergoing another reexamination of the value and relevance of peer review as a vehicle for the optimal evaluation and transmission of new findings and knowledge.

Peer review and the process by which medical journals select manuscripts to publish has always been subject to criticism. It is often said that the external reviewers are the biggest variable in how an article is graded. Reviewers differ in how critical they are, the topics in which they are interested, and potential rivalry with or admiration of other investigators. An examination that I performed previously for another publication revealed that nearly a quarter of manuscripts were graded in the highest categories by one reviewer and in the lowest by another. Even when the critiques of two reviewers were nearly identical, their grades sometimes varied substantially. As discussed above, even when a paper is accepted, the time required for review and publication is often very lengthy and delays the diffusion of information.

The emergence of online medical journals has addressed some of the above limitations. Papers can be posted online quickly after acceptance, reducing to some extent the time required for publication. The internet provides a venue by which commentaries by readers can be rapidly transmitted. The absence of the expense of printing also conveys the potential to increase the number of articles published. It is not surprising that there has been a progressive move of journals and readers to online publications.

Although clearly of value, online publication of medical journals has begotten a troublesome problem. A number of online only periodicals have arisen, sometimes referred to as predatory publications, that seem to exist primarily to generate a profit. These journals have been characterized by weak or seemingly nonexistent peer review and sometimes with ostensibly absent or limited editorial boards. A fee is charged to publish a paper which, given the cost of posting online, can generate a considerable profit. Such publications do provide a venue for articles that have not found acceptance. However, they have the potential to publish inaccurate information which may result in even greater damage than preprint servers since the publication conveys a greater sense of validity.

A longstanding alternate approach to remedy the delay in publication of new findings by journals has been presentation of data at medical meetings. Similar to preprint servers there is cursory review at best, while in contrast to preprints the information is available only to those who attend the meeting. In addition, there is typically no archiving of the presentation. Advocates point out that important medical meetings are now nearly always covered in detail online by a variety of sources that present not only the results but often also critiques by authorities. Thus, some have maintained that the information presented and reviewed at medical meetings is an adequate substitute for peer review, and that the results reported at meetings and as reviewed online is actionable. This requires, of course, that one accepts the accuracy and interpretation of the reporter.

In aggregate, existing limitations coupled with the availability of alternate information sources has again raised the question of the value and relevance of medical journals. As any editor clearly knows, and as delineated above, medical journals have many imperfections. However, passing the crucible of peer review is the best assurance that new information is valid and may be actionable. So, like democracy, peer review journals may be imperfect, but they are the best alternative available. While providing some advantages, preprint servers and other sources for new investigative results all have significant limitations as well. They are perhaps most valuable in defining the areas in which current medical journals need to improve. In my view, the alternate sources of medical information will remain an important supplement to journals. However, they will never replace them. Since its initiation, peer review has survived for 360 years. It continues to be the most accurate and reliable method to assess new research findings, and surely will not become obsolete in the foreseeable future.

## Funding

The author has no funding to report.

## Disclosure Statement

The author reports no conflict of interest.
